# Prevalence of newly detected diabetes in pregnancy in Qatar, using universal screening

**DOI:** 10.1371/journal.pone.0201247

**Published:** 2018-08-03

**Authors:** Mohammed Bashir, Manar E. Abdel-Rahman, Mahmoud Aboulfotouh, Fatin Eltaher, Khalid Omar, Isaac Babarinsa, Kwabena Appiah-Sakyi, Tarek Sharaf, Eman Azzam, Mohammad Abukhalil, Malika Boumedjane, Wigdan Yousif, Warda Ahmed, Sadaf Khan, Justin C. Konje, Abdul-Badi Abou-Samra

**Affiliations:** 1 Qatar Metabolic Institute, Endocrine Department, Hamad Medical Corporation, Doha, Qatar; 2 Department of Biostatistics, College of Health Sciences, Qatar University, Doha, Qatar; 3 Department of Obstetrics and Gynaecology, Women’s Hospital, Hamad Medical Corporation, Doha, Qatar; 4 Department of Obstetrics and Gynaecology, Faculty of Medicine, Minia University-Minia, Egypt; 5 Department of Obstetrics and Gynaecology, Sidra Medical and Research Centre-Doha, Qatar; Dalian University Affiliated Xinhua Hospital, CHINA

## Abstract

**Background:**

Diabetes first detected during pregnancy is currently divided into gestational diabetes mellitus (GDM) and diabetes mellitus (DM)- most of which are type 2 DM (T2DM). This study aims to define the prevalence and outcomes of diabetes first detected in pregnancy based on 75-gram oral glucose tolerance test (OGTT)using the recent WHO/IADPSG guidelines in a high-risk population.

**Methods:**

This is a retrospective study that included all patients who underwent a 75 g (OGTT) between Jan 2016 and Apr 2016 and excluded patients with known pre-conception diabetes.

**Results:**

The overall prevalence of newly detected diabetes in pregnancy among the 2000 patients who fulfilled the inclusion/exclusion criteria was 24.0% (95% CI 22.1–25.9) of which T2DM was 2.5% (95% CI 1.9–3.3), and GDM was 21.5% (95% CI 19.7–23.3). The prevalence of newly detected diabetes in pregnancy was similar among the different ethnic groups.

The T2DM group was older (mean age in years was 34 ±5.7 vs 31.7±5.7 vs 29.7 ±5.7, p<0.001); and has a higher mean BMI (32.4±6.4 kg/m2 vs 31.7±6.2 kg/m2 vs 29.7± 6.2 kg/m2, p< 0.01) than the GDM and the non-DM groups, respectively. The frequency of pre-eclampsia, pre-term delivery, Caesarean-section, macrosomia, LGA and neonatal ICU admissions were significantly higher in the T2DM group compared to GDM and non-DM groups.

**Conclusion:**

Diabetes first detected in pregnancy is equally prevalent among the various ethnic groups residing in Qatar. Newly detected T2DM carries a higher risk of poor pregnancy outcomes; stressing the importance of proper classification of cases of newly detected diabetes in pregnancy.

## Introduction

Diabetes first detected during pregnancy is classified as gestational diabetes mellitus (GDM) and type 2 diabetes mellitus (T2DM)[1]. GDM is associated with significant short and long-term sequelae on both the mother and the offspring. During pregnancy, gestational diabetes is associated with increased risk of pre-eclampsia, pre-term labour, Caesarean-section, macrosomia, shoulder dystocia, together with a substantial increase in medical cost [2][3]. Post-delivery, mothers with GDM have a 7–13 folds increase risk of progression to T2DM and are at increased risk for other cardio-metabolic disorders compared to mothers with normoglycaemia during pregnancy [4][5]. Offspring born to mothers with GDM are at increased risk of obesity, T2DM and other cardio-metabolic abnormalities compared to offspring of normoglycaemic mothers [6][7].

There are not too many areas of diabetes that have generated as much debate, controversy, and lack of consensus as newly detected DM in pregnancy. The debates cover the diagnostic criteria, classification, the timing of screening, and the method of screening (universal versus selective screening) [1][8][9][10]. It is therefore not surprising that the prevalence of newly detected diabetes in pregnancy varies according to the detection method. This, indeed, is the single most probable explanation for the wide variations in the prevalence of newly detected diabetes in pregnancy both globally and within the same country [11][12][13].

Qatar is a growing urbanised country in the Arabian Gulf with a high prevalence of obesity and T2DM among child-bearing age women[14]. Most of the long-term expats come from countries with high prevalence of T2DM too; such as the Middle East, North Africa, and the Indian sub-content. A 2011 study reported that the prevalence of GDM in Qatar was 16.3%[15]. The study used selective screening for detecting GDM. The Qatar Stepwise report has shown that the prevalence of diabetes in Qatar is 16.7% and that 70.1% of the population in Qatar are either overweight or obese[14]. Due to this high prevalence of diabetes and obesity, Qatar has implemented a universal screening approach for all pregnancies. It has also adopted the WHO & International Association of the Diabetes and Pregnancy Study Groups (IADPSG)-75 gram OGTT diagnostic criteria. Globally, the implementation of this new diagnostic criteria is believed to have increased the prevalence of newly detected diabetes in pregnancy by 2–3 folds with a concordant increase in demand on health care services [16]. Our aim was, therefore, to determine the current prevalence of newly detected diabetes in pregnancy in our population based on universal screening strategy.

## Methods

This is a retrospective, cross-sectional study, which was undertaken at the Women’s Hospital, Hamad Medical Corporation in Doha. The Women’s Hospital is the largest maternity hospital in Qatar overlooking 16–18 thousand deliveries per annum.

### Universal screening for diabetes in pregnancy in the State of Qatar

The antenatal care is delivered in the Primary Health Care Corporation and Hamad Medical Corporation. Both, primary and secondary care systems are using the same electronic medical records-Cerner. Each patient has one universal identification number. All pregnant women are screened in the first ANC visit using fasting blood glucose and HBA1c- to rule out pre-existing diabetes. Then 75 grams OGTT is performed between 24–32 weeks gestation in low-risk patients and between 16–20 weeks gestation in high-risk patients (obese, PCOS, previous history of GDM, previous history of Intra-uterine fetal death, previous history of a macrosomic baby). Pre-existing diabetes is diagnosed based on one or more abnormal glucose values (fasting blood glucose ≥ 7.0 mmol/l, 2-hours post 75 grams OGTT ≥11.1 mmol/ l; and HBA1C ≥ 6.5%). As most of these patients are asymptomatic and are detected during routine screening, they are labelled as T2DM. Gestation diabetes (GDM)is diagnosed based on based on one or more abnormal glucose values; (fasting blood glucose ≥ 5.1 mmol/l; 1-hour post 75 grams OGTT ≥10.0 mmol/ l; and 2-hours post 75 grams OGTT ≥ 8.5 mmol/l [1].

### Study population

For the purpose of this study, we included all women who underwent 75 grams OGTT between January 2016 and April 2016 in the Women’s hospital. Subjects were identified using the laboratory database. Subjects who did not complete the full two hours OGTT were excluded from the study-unless the fasting blood glucose was diagnostic of diabetes in pregnancy. Subjects who were known to have pre-conception diabetes were excluded from the study. Subjects were classified into Normal, GDM or T2DM based on the WHO criteria as detailed above.

We screened 2300 subjects, of which 2000 subjects met the selection criteria and were included in the final analysis of the prevalence of diabetes in pregnancy. In the maternal and neonatal outcomes section; we excluded 122 patients from the analysis due to missing delivery data. All maternal and neonatal data were collected from the electronic medical record-Cerner. Patient’s ethnicity was classified into Qatari; non-Qatari Arab (residents of the Middle East and North Africa Region); Asian (residents from the India sub-continent and the Philippines) and Others. Pre-pregnancy weight is recorded in the first visit based on patient self-report and is entered into the electronic medical records as ‘‘ pre-pregnancy weight”. If this was not recorded, we used the last recorded weight before conception as pre-pregnancy weight otherwise the weight is considered to be missing. We used the last height recorded before conception or the height recorded in the first antenatal visit to calculate the BMI. Maternal age was calculated as the age of the mother at conception. Macrosomia is defined as birth weight > 4000 grams and LBW (low birth weight) is defined as birth weight <2500 grams. LGA (Large for gestational age) is defined as birth weight >90^th^ percentile nd SGA (small for gestational age) is defined as birth weight <10^th^ percentile using the locally adapted growth charts. Pre-term delivery is defined as delivery < 37 weeks’ gestation. Hypertensive disorders in pregnancy were diagnosed based on the American College of Obstetrics and Gynaecology guidelines[17]. The study was approved by the Institutional Review Board at Hamad Medical corporation.

### Statistical analysis

Statistical analysis was performed using STATA 14 software (College Station, TX: Stata Corp LP). Data was thoroughly checked and cleaned using ranges, missing values and internal consistency, returning to original data source when needed. Categorical variables were expressed as a percentage (%) and mean ± standard deviation for normally distributed continuous variables. Prevalence was expressed as a percentage (%) with 95% logit confidence interval (CI). Analysis of variance (ANOVA) was used to compare continues variables between the three groups. Test of linear effect after ANOVA to assess the linear trend of means across groups. Chi-square test was used to compare categorical variables. P value < 0.05 was considered significant

## Results

Two thousand patients (2000) were included in the study. Of those 754 (37.7%) were Qatari, 705 (35.25%) were non-Qatari Arabs and 446 (22.3%) were Asians and 95 (4.75%) were Others. As shown in Table 1, 70.7% of the cohort were either obese or overweight. The non-Qatari Arabs cohort had the highest prevalence of obesity, (50%) followed by the Qataris (46%).

**Table 1:** Baseline characteristics of study subjects.

	Qatari	Arab	Asian	Other	Total
Age in years*(mean±SD)	29.8±5.9	30.3±5.6	30.4±5.0	32.0±5.8	30.2±5.6
Age in years* **n(%)**					
**< = 20**	24(3.2%)	19(2.7%)	6(1.3%)	1(1.1%)	50(2.5%)
**>20–25**	147(19.5%)	108(15.3%)	55(12.3%)	11(11.6%)	321(16.1%)
**>25–30**	222(29.4%)	224(31.8%)	160(35.9%)	26(27.4%)	632(31.6%)
**>30–35**	210(27.9%)	212(30.1%)	143(32.1%)	25(26.3%)	590(29.5%)
**>35–40**	118(15.6%)	103(14.6%)	62(13.9%)	22(23.2%)	305(15.3%)
**>40–51**	33(4.4%)	39(5.5%)	19(4.3%)	10(10.5%)	101(5.1%)
**Total**	**754(100%)**	**705(100%)**	**445(100%)**	**95(100%)**	
**BMI (kg/m^2^)** ** **(mean±SD)**	30.0±6.8	30.4±6.2	27.5±5.3	28.5±6.3	29.5±6.4
BMI categories** **n(%)**					
**Underweight (<18)**	2(0.3%)	2(0.3%)	1(0.2%)	0(0.0%)	5(0.3%)
**Normal (18–24.9)**	162(21.5%)	131(18.6%)	142(31.8%)	29(30.5%)	464(23.2%)
**Overweight (25–29.9)**	206(27.3%)	214(30.4%)	170(38.1%)	30(31.6%)	620(31.0%)
**Obese 1 (30–34.9)**	215(28.5%)	178(25.2%)	72(16.1%)	18(18.9%)	483(24.1%)
**Obese 2 (35–39.9)**	92(12.2%)	105(14.9%)	28(6.3%)	8(8.4%)	233(11.7%)
**Obese 3(≥ 40)**	40(5.3%)	44(6.2%)	9(2.0%)	5(5.3%)	98(4.9%)
**Total**	**754(100%)**	**705(100%)**	**446(100%)**	**95(100%)**	**2000(100%)**

* Age has 0.05% missing observation

** BMI and weight have 4.9% missing observations

Table 2 shows that the overall prevalence of newly detected diabetes in pregnancy was 24.0% (95% CI 22.1–25.9); 2.5% (95% CI 1.9–3.3) was T2DM, and 21.5% (95% CI 19.7–23.3) was GDM. Among the Qatari population, the prevalence of newly detected diabetes in pregnancy was 25.8% (95% CI 22.7–29.0); 2.3% (95% CI 1.4–3.6) was T2DM, and 23.5% (95% CI 20.6–26.6) was GDM.

**Table 2:** Prevalence of newly discovered diabetes in pregnancy expressed in number, percentage and 95% CI.

Ethnicity	T2DM		GDM		Total	
	N	Percent (95% CI)	N	Percent (95% CI)	N	Percent (95% CI)
**Qatari**	17	2.3% (1.4,3.6)	177	23.5% (20.6,26.6)	194	25.8% (22.7,29.0)
**Arab**	18	2.6% (1.6,4.0)	137	19.4% (16.7,22.5)	155	22.0% (19.1, 25.2)
**Asian**	12	2.7% (1.5,4.7)	97	21.7% (18.2,25.8)	109	24.4% (20.7,28.7)
**Other**	3	3.2% (1.0,9.5)	18	18.9% (12.2,28.3)	21	22.1% (14.8, 31.6)
**Total**	50	2.5% (1.9,3.3)]	429	21.5% (19.7,23.3)	479	24.0% (22.1,25.9)

As shown in Table 3, there was significant difference in age and BMI between the three groups. Pre-eclampsia was more common in those with T2DM compared to those with GDM and non-DM while the rates of pregnancy-induced hypertension were not different between the three groups(Table 3). Pre-term labour and Caesarean-section were more common in patients with T2DM and GDM groups compared to non-DM group while the frequency of polyhydramnios was similar across the three groups.

**Table 3:** Maternal and fetal outcomes according to newly discovered DM in pregnancy.

	T2DM	GDM	Normal	p-value
	(n = 47)	(n = 402)	(n = 1,429)	
Age in years (mean±sd)*	34.3±5.7	31.7±5.6	29.7±5.6	**<0.001**
**BMI (kg/m**^**2**^**) (mean±sd)**	32.4±6.5	31.7±6.3	28.8±6.3	**<0.001**
BMI categories**				**<0.001**
**< 18**	0 (0.0%)	0 (0.0%)	5 (0.4%)	
**18–24.9**	5 (11.1%)	46 (12.0%)	381 (27.8%)	
**25–29.9**	12 (26.7%)	116 (30.3%)	465 (33.9%)	
**30–34.9**	13 (28.9%)	120 (31.3%)	321 (23.4%)	
**35–39.9**	11 (24.4%)	68 (17.8%)	143 (10.4%)	
**≥ 40**	4 (8.9%)	33 (8.6%)	56 (4.1%)	
Pregnancy induced hypertension*	2 (4.4%)	18 (4.5%)	32(97.5%)	**0.166**
**Pre-eclampsia**	5 (10.6%)	11 (2.8%)	36 (2.5%)	**0.004**
Recurrent Vaginal infections*	1 (2.1%)	7 (1.8%)	23 (1.6%)	**0.951**
**Recurrent UTI**	3 (6.4%)	18 (4.5%)	66 (4.6%)	**0.845**
**Preterm delivery**	8 (17.4%)	69 (17.2%)	157 (11.1%)	**0.003**
**Induction of Labour**	8 (17.0%)	61 (15.3%)	149 (10.6%)	**0.017**
**Mode of Delivery**				**<0.001**
**Vaginal delivery**	17 (37.8%)	232 (58.3%)	920 (63.8%)	
**Caesarean-section**	28 (62.2%)	166 (41.7%)	499(35.2%)	
**Neonatal weight kg (mean±sd)**	3.14±0.8	3.16±0.7	3.15±0.6	**0.958**
**Neonatal weight percentile**				**<0.001**
**SGA**	4 (8.7%)	44 (11.0%)	202 (14.2%)	
**AGA**	33 (71.7%)	300 (75.2%)	1,122 (79.0%)	
**LGA**	9 (19.6%)	55 (13.8%)	96 (6.8%)	
**Neonatal birth weight categories**				**<0.001**
**Low**	8 (17.4%)	52 (13.0%)	137 (9.6%)	
**Macrosomia**	4 (8.7%)	37 (9.3%)	60 (4.2%)	
**Normal**	33(73.3%)	308(77.6%)	1217(86.2%)	
**Neonatal outcome**				**0.576**
**Life Birth**	47(100%)	396(98.5%)	1417(99.2%)	
**Miscarriage**	0 (0.0%)	1 (0.25%)	0 (0.0%)	
**Neonatal Death**	0 (0.0%)	1 (0.25%)	3 (0.2%)	
**Still Birth**	0 (0.0%)	4 (1.0%)	9 (0.6%)	
**NICU admission**†	10 (21.7%)	58 (14.7%)	114 (8.1%)	**<0.001**
**Respiratory distress**†	9 (19.6%)	32 (8.1%)	67 (4.7%)	**<0.001**
**Shoulder Dystocia**†	0 (0.0%)	2 (0.5%)	4 (0.3%)	**0.729**
**Neonatal Hypoglycaemia**†	13 (28.3%)	45 (11.3%)	39 (2.7%)	**<0.001**
**Polycythaemia**†	1 (2.2%)	3 (0.8%)	5 (0.4%)	**0.146**
**Jaundice**†	9 (19.6%)	50 (12.7%)	137 (9.7%)	**0.030**

*< 1% missing,

**<4% missing,

^†^<2% missing

There were no differences in the mean birth weight in the three groups. However, the frequency of LGA infants was higher in the T2DM compared to the GDM and Non-DM groups (19.6% vs 13.8% vs 6.8% respectively; p< 0.001). The frequency of macrosomia was similar in T2DM and GDM groups but higher than in the non-DM group (8.7% vs 9.3% vs 4.2% respectively; p<0.01). There were no significant differences in the frequencies of stillbirth and neonatal death between the three groups. The rates of Neonatal Intensive Care Unit (NICU) admissions were significantly higher in the T2DM group compared to the GDM and non-DM group (21.7% vs 14.7% vs 8.1% respectively, p<0.001)

Most of the patients with newly detected diabetes in pregnancy were managed with diet alone, 84.8% (364) in the GDM group and 52% (26) in the T2DM group. Among those who needed treatment in the GDM group; 54.8% were treated with Metformin, 37% with Metformin and insulin and 8.2% with insulin alone. In patients with T2DM who required treatment, 29.3% were treated with Metformin alone, 54% with Metformin and insulin and 16.8% were treated with insulin alone.

## Discussion

This is the first study from the MENA (the Middle East and North Africa) area to report on the prevalence of newly detected diabetes in pregnancy based on universal screening. The prevalence of newly detected diabetes in pregnancy is 24.0%, of which 89.6% have GDM, and 10.4% have newly discovered T2DM. The prevalence of newly detected diabetes in pregnancy in the Qatari population was 25.8%; 2.3% had T2DM, and 23.5% had GDM. Patients with newly detected diabetes in pregnancy were older and more likely to be obese compared to the background population. The frequencies of maternal complications; pre-eclampsia, induction of labour, pre-term labour and Caesarean-section were higher among the diabetes group compared to the background population. Similarly, the frequencies of neonatal complications; LGA, macrosomia, NICU admission, respiratory distress, neonatal hypoglycaemia and neonatal jaundice; were higher among the diabetes group compared to the background population.

As previously stated, the comparison of the prevalence of newly detected diabetes in pregnancy between centres is problematic due to the variations in the detection methods. Our results are similar to the IDF report from 2014 that estimates the prevalence of newly detected diabetes in pregnancy in Qatar to be 25.4% [13]. Our study results are also similar to a large registry data from Saudi Arabia; The Riyadh Mother and Baby Multicentre Cohort Study; which reported a GDM prevalence of 24% among Saudi women [18]. On the other hand, Alfadhli et al., from Saudia Arabia, reported a GDM prevalence of 39.4% based on universal OGTT screening performed in 444 patients at 24–28 weeks’ gestation [11]. While Al-Serehi et al.,reported a 13% prevalence of GDM after studying records of 1718 Saudi women; the method of detection was not clear[12]. Studies from Oman and Bahrain reported a lower prevalence of GDM; 10% in both countries [19][20].

In 2011, Bener et al. followed 1608 pregnancies and reported a GDM prevalence of 16.3% in Qatar; yet the study has combined both GDM and T2DM under one category[15]. The screening with OGTT was selective based on the presence of one or more risk factor for GDM and a random blood glucose >7.7 mmol/l. Besides, 75 grams OGTT was used for screening but the criteria that were used to diagnose GDM was not outlined. Due to the short interval between the previous study and ours; we believe that the 46.6% increase in the prevalence of newly detected diabetes in pregnancy is due to the implementation of universal screening and the adoption of the WHO rather than an absolute increase in the prevalence of diabetes in pregnancy in Qatar. The prevalence of overweight and obesity in the background population was 71.8% which is similar to a previous report from Qatar that shows that the 70.1% of the Qatari population are either overweight or obese [14]. While this paper does not focus on identifying risk factors for diabetes in pregnancy, the high risk of overweight and obesity in Qatar is indeed a likely major contributing factor to the high prevalence of diabetes in pregnancy.

The rates of maternal and fetal complications reported in our study are similar to other countries [21][22][23][24]. The study by Bener et al. classified all patients with newly detected diabetes in pregnancy as GDM; hence it will not be feasible to compare the two cohorts together [15]. Despite the high prevalence of diabetes in pregnancy in Qatar, 84.8% of the GDM patients did not require pharmacotherapy. This is in line with results from two randomised control trials in which 80% and 92% of patients with GDM were managed on a diet alone, respectively[25][26].

Being retrospective is the main limitation of this study. Furthermore, currently, all pregnant ladies are screened at the first ANC visit using fasting blood glucose and HBA1c. It is therefore likely that those with impaired fasting blood glucose would have been identified during the first visit and will therefore not have undergone OGTT screening and would not have featured in our cohort. Knowing that the prevalence of impaired fasting glucose among females between 18–44 years of age is 6.2%, the overall prevalence of newly detected in Qatar could be as high as 30%[14]. Prospective studies are needed to provide more exact data. Anti-GAD antibodies and anti-islet cells antibodies were not performed and hence there is a remote possibility that some of the T2DM have actually type 1 DM.

The impact of GDM on mothers and offspring extend beyond the three trimesters. GDM is known to increase the progression of the mother to T2DM at a young age[27]. There is evidence that this rate of progression has increased over the last two decades[28]. *In-utero* exposure to hyperglycaemia increases the risk of obesity and early onset T2DM in the offspring. Sellers et al. showed that the risk of developing T2DM is increased by 3–4 folds in children born to mothers who had GDM with a cumulative incidence of 15% by the age of 30 years[29]. Similar data from Pima Indians showed that, by 30 years of age, almost 30% of the offspring born to mothers who had GDM, have developed T2DM[30]. Qatar has one of the highest rates of T2DM in the world especially among those under 45 years of age (11.8% in both genders)[14]. In addition to the genetic and environmental factors, we believe that the high rate of hyperglycaemia during pregnancy is an important contributor to the high prevalence of early-onset T2DM. Efforts to reduce the prevalence of T2DM in Qatar and other MENA countries should include measures to prevent GDM and to prevent progression to T2DM post-delivery.

## Supporting information

S1 Data (XLSX)
